# 150 Years of the Mass Action Law

**DOI:** 10.1371/journal.pcbi.1004012

**Published:** 2015-01-08

**Authors:** Eberhard O. Voit, Harald A. Martens, Stig W. Omholt

**Affiliations:** 1 Wallace H. Coulter Department of Biomedical Engineering, Georgia Tech and Emory University, Atlanta, Georgia, United States of America; 2 Department of Engineering Cybernetics, Norwegian University of Science & Technology, Trondheim, Norway; National Cancer Institute, United States of America and Tel Aviv University, Israel, United States of America

## Abstract

This year we celebrate the 150th anniversary of the law of mass action. This law is often assumed to have been “there” forever, but it has its own history, background, and a definite starting point. The law has had an impact on chemistry, biochemistry, biomathematics, and systems biology that is difficult to overestimate. It is easily recognized that it is the direct basis for computational enzyme kinetics, ecological systems models, and models for the spread of diseases. The article reviews the explicit and implicit role of the law of mass action in systems biology and reveals how the original, more general formulation of the law emerged one hundred years later ab initio as a very general, canonical representation of biological processes.

## Introduction

Some things not only seem timeless, they also seem to have been with mankind forever. Apple pies, sandals, and drums come to mind. In the field of computational chemistry and biochemistry, the same attribute appears to apply to the law of mass action. This law describes how a chemical reaction takes place under ideal conditions. Namely, if a substance A reacts with substance B to form substance C, then the production of C follows the function:
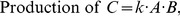
(1)where A, B, and C are concentrations that typically change over time until the reaction comes to an end, and *k* is a rate constant describing the speed of the reaction. This formulation of a fundamental process seems so natural and intuitive today that only historians seem to remember its beginnings, and hardly anyone cites its originators. Nonetheless, the perception of the mass action law's eternal existence is just that, a perception.

It is 150 years this year that the seminal work of two Norwegians, Cato Maximilian Guldberg and Peter Waage, was first published. A few years earlier, fate had brought them together; they worked together and ultimately even became brothers-in-law. Guldberg (1836–1902) was a mathematician, to whom Norway's crown prince had just awarded a gold medal for solving a challenge problem related to the manner in which circles touch. Waage (1833–1900) was a chemist, who had also received the crown prince's gold medal, for an essay on the theory of radicals in acids containing oxygen. Guldberg at the time held a teaching appointment at the Norwegian Royal Military University, and Waage had just been named Professor of Chemistry at Oslo University, which at the time was called Christiana University [1]. The two started to collaborate in 1862 and documented the law of mass action in a series of three articles [2]–[4]. The first of these was published in Norwegian in 1864 (for an English translation see [5]), and the second in French in 1867. Primarily due to the choice of these languages, both articles remained essentially unknown until Jacobus Henricus van't Hoff in 1877 developed similar ideas [6], and Guldberg and Waage published their third paper in 1879 in German, the language of chemistry at the time.

Of course, Guldberg and Waage did not operate in a vacuum. Chemists had been thinking about the affinity between molecules for quite some time. In a seminal treatise of 1775 [7], the Swedish geochemist Torbern Olof Bergman (1735–1784) postulated that if substance A had a stronger attraction for substance B than for substance C, then B would displace C from its complex with A, so that AC+B→AB+C. This simple idea allowed Bergman to establish a table in which numerous reactions were ranked by the strengths of their affinities. Bergman's work had a strong impact on the manner in which affinity was pondered at the end of the 18th century. Importantly, affinity was considered a genuine feature of every substance, and chemical reversibility was not permitted, because it violated the perceived order of affinities, as documented in Bergman's tables [8]. In truth, some reversible reactions had been observed, but they did not fit the prevalent thinking of the time and were therefore deemed anomalous and due to peculiar conditions.

Dissatisfied with the concept of affinity and its flaws, the French chemist Claude Louis Comte Berthollet (1748–1822) developed, between 1801 and 1803, an opposing theory that permitted reversibility [9]–[11]. According to his new theory, all substances had an affinity toward each other, but its degree depended on the properties of each substance, such as its solubility. Moreover, Berthollet never considered a displacement reaction complete: if A had an affinity for B and for C, and B and C were in excess, one would always find a distribution of AB, AC, B, and C. This distribution was explained with a balance between opposing forces, whose strengths depended on the relative affinities as well as the quantitative proportions of the substances [3]. Berthollet's theory also had some problems [12], [13], and it was conceptual rather than formalized, let alone mathematical.

The French organic chemist and early thermodynamicist Henri Victor Regnault (1810–1878) discussed another problem with the current concept of affinity by demonstrating with careful experiments that steam oxidizes metals at certain temperatures, but that these oxides are reduced by steam at other temperatures [14]. He further showed that both reactions could happen simultaneously. These experiments provided proof that the difference between two forces alone was not sufficient for explaining a chemical reaction, and that the amounts of substances, as well as other factors, had to be determinants of chemical reactions. Quite a different approach to understanding chemical reactions was chosen by the proponents of “Wärmetheorie” (“heat theory”), which one may consider a precursor of thermodynamics [3], [15]. According to this way of thinking, every molecule, A, had a specific heat, *f*(A). The theory said that two substances A and B could only form a product if *f*(A)+*f*(B)>*f*(AB) [3].

Guldberg and Waage doubted the generality of this theory, because experience had shown that the experimental conditions determined whether a reaction could take place at all and whether it generated or consumed heat. At the same time, they disagreed with Berthollet's theory, recognizing that the amounts relative to each other and to the reaction volume determined the progression of a reaction. Formalizing these insights, Guldberg and Waage focused on the chemical equilibrium as well as the rate of the reaction.

Guldberg and Waage were particularly intrigued by a new experimental approach to studying ester reactions, which occurred at much slower rates than other standard reactions of the time. This new approach had just been reported during 1862–1863 by Berthelot and Saint-Gilles [16], [17] and helped them demonstrate that at any point in time the amount of ester formed was proportional to both the alcohol and acid concentrations. The experiments also showed that the reaction remained incomplete, and that both substrates and both products were always available. Berthelot and Saint-Gilles formulated the forward process mathematically, but never considered the reverse reaction between ester and water [8]. In an unrelated study, which in retrospect indicates that the time was ripe for new, formal ideas and concepts about affinity, Ludwig Wilhelmy derived a differential mass action equation for the inversion of cane sugar [18], [19], although Guldberg and Waage were apparently not aware of this study.

Intrigued by the new experimental options developed by Berthelot and Saint-Gilles, Guldberg and Waage performed 300 experiments that investigated reversible reactions [3]. They motivated this effort as follows: “The theories that so far have been proffered in chemistry with regard to the action of chemical forces are deemed unsatisfactory by all chemists. This is true for electrochemical as well as thermochemical theories; indeed, it seems doubtful whether it will ever be possible to discover the laws with which chemical forces act, based on the generation of electricity and heat that accompany chemical processes. We have therefore attempted to find a more direct method for determining the mode of action of these forces, and we believe to have found, with our quantitative investigation of the mutual interactions of different substances, a way that will most safely and naturally reach the goal” (translated from [1], [2]).

The key to Guldberg and Waage's theory was thus the balance between two forces that are simultaneously in effect during a chemical reaction, one that joins and one that disassembles. In fact, they adamantly declared it imperative to account for both forces simultaneously, if a realistic quantitative description was to be found. Their strategy was therefore to characterize these forces mathematically, and then to formulate a general law that would encapsulate their balance, but also account for modulation of these forces, for instance, by temperature and solubility. Quasi as a special case, the two forces could be equally strong, and if so, the system had reached a steady state, which was always the same if the conditions were the same.

Thus, in their 1867 and 1879 papers [3], [4], the force of a reaction between two substances with *M* and *N* molecules, respectively, and occurring in a volume, *V*, was formulated as
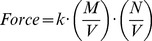
(2)Considering concentrations instead of numbers of molecules, a generic reaction with two substrates, *p* and *q*, and two products, *p*′ and *q*′, was therefore formulated as
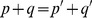
(3)According to Equation 2, the forces in this reversible reaction are *k p q* and *k*′ *p*′ *q*′, respectively. Rewriting the rate parameters as *k*′ = α *k*, the steady state in this system is reached if the forces are balanced, that is, if
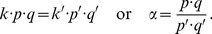
(4)Guldberg and Waage called this state a “mobile steady state,” because it balances two dynamic forces that simultaneously occur in opposite directions and eventually equilibrate in this steady state. To formalize this dynamics, Guldberg and Waage assumed as the starting point an arbitrary state and argued that a certain amount of material, *x*, had to be converted during the approach of the mobile steady state. Thus, for every molecule of type *p* undergoing the reaction, one molecule of type *q* was lost and, at the same time, one molecule of type *p*′ and one molecule of type *q*′ were gained. Accounting for the fact that reactions occurred simultaneously in both directions, they formulated the dynamics as
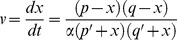
(5)
[2], [3]. The forces became equal at the steady state, where *v* = 0. Guldberg and Waage noted explicitly [3] that if one could measure *p*, *q*, *p*′, and *q*′, then one could determine the ratio between the affinity coefficients, α = *k′*/*k*, and that, conversely, knowledge of α allowed predictions regarding the reaction. They subsequently expanded the same ideas for reactions requiring, for instance, two molecules of substance M and one molecule of N, and argued that the rate expression naturally emerges as *k M* ^2^ *N*.

The mass action law was much later derived by others from first principles of equilibrium thermodynamics, balanced potentials, statistical mechanics, equilibria in multicomponent systems, and mean field approximations [20], [21]. As an example, Kittel and Kroemer [22], and Kudriavtsev et al. [23], demonstrated how the mass action law can be derived from chemical potentials or statistically independent, interacting molecules in ideal gases under constant pressure and temperature. Other authors discussed the deterministic mass action concepts in the context of stochastic processes [24], [25]. These newer derivations do not diminish the value and enormous applicability of Guldberg and Waage's conjecture. On the contrary, they highlight how fundamental, as well as chemically and physically meaningful, the law of mass action is.

In addition to various derivations of the mass action law, different generalizations were proposed over the years. In particular, several authors discussed time-dependent rate constants or suggested real-valued kinetic orders, especially for species in reactions that are constrained to one or two dimensions or occur under conditions of molecular crowding, where the best process representation appears to be fractal kinetics [26]–[29].

## Impact of the Mass Action Law on Computational Systems Biology

Guldberg and Waage's intuition and mathematical formulation may have been all but forgotten by many, but the impact of the mass action law on the fields of chemistry, biochemistry, biology, pathway analysis, and systems biology can hardly be overstated. To appreciate this impact, it is useful to study how mathematical models are designed in modern-day systems biology.

Computational systems biology uses mathematical and computational methods in an attempt to understand how biology works, with a secondary goal of manipulating and optimizing biomedical systems, guided by mathematics. Efforts are also increasing to improve clinical applications by biomedical modelling. The key to these investigations is a valid mathematical model. While novices often believe that the mathematical analysis of such a model is the most complicated part of biological systems analysis, the truly more difficult aspect is the choice and design of the model. After all, biological systems are complicated, and nature has not provided us with guidelines telling us how to translate a biological phenomenon into the optimal, or at least an appropriate, computable structure. One might assert that biological processes must obey the laws of physics, which would dictate the best formulation, and while this assertion is certainly true, many biological processes are convoluted systems of numerous elementary steps. Thus, a full mechanistic description is beyond the capacity of today's science, in terms of mathematical details, parameter identification, and computational power, as well as our cognitive capacity.

Consider, for example, the expression of a gene in response to a chemical stimulus, such as a hormone. The hormone reaches a target cell and binds to the external portion of a cellular receptor, which penetrates the cell membrane. The binding event typically causes a conformational change of the internal portion of the receptor protein, which in turn causes a number of molecular events that ultimately trigger a signaling cascade. This cascade consists of several layers, in which specific proteins are phosphorylated. This phosphorylation may actually cross over onto other signaling cascades, but the main goal is an amplified, denoised signal that, for instance, may lead to the translocation of a transcription factor from the cytosol into the nucleus, where it binds to a specific region of DNA, which ultimately leads to a change in gene expression. A molecular biologist will cringe at the simplifications in this description of events, but it seems quite evident that even this abridged model would be extremely difficult to translate into elemental physical processes.

More generically, a modeler must ask whether “true” mathematical representations even exist and, if not, what to do about it. To address this substantial challenge, systems biology either assumes particular mathematical formulations to be valid or resorts to approximations. In fact, even the former strategy is not all that different from the latter, because it must ultimately employ approximations. As it turns out, the approximations used in systems biology typically lead to the law of mass action. A few representative examples will suffice to demonstrate the pervasiveness of this formulation.

**Figure 1:**
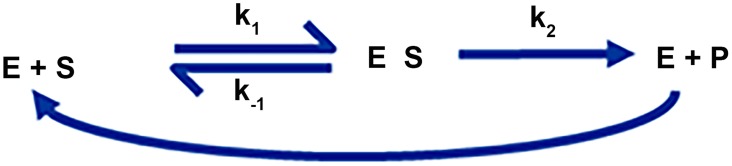
Diagram of a typical Michaelis-Menten reaction, in which an enzyme, E, catalyzes the conversion of a substrate, S, into a product, P, via the formation of an intermediate complex, ES. The indexed quantities, *k*, denote reaction rates.

The most prevalent model in biochemistry is the Michaelis-Menten rate law (MMRL) [30]

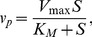
(6)which describes a single-substrate, enzyme-catalyzed reaction that may be diagrammed as shown in Fig. 1. The format of MMRL in Equation 6 is the result of a useful approximation of a system of three differential equations, namely,
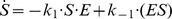


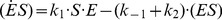
(7)

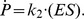
In this direct mathematical formulation of the diagram in Fig. 1, all terms follow the mass action format developed by Guldberg and Waage [3]. Even if inhibitors are included in the process, the same model design strategies hold, and one either obtains a formulation corresponding to the approximation in Equation 6 or a set of differential equations that again exclusively contain processes in mass action format [31].

A slightly more complicated example is a reversible bi-substrate reaction of the type
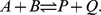
On the surface, one might use two mass action laws, but if an enzyme is involved, the elemental reaction scheme becomes unwieldy. According to Schultz [32], an appropriate model for the rate, *v*, of the reaction is
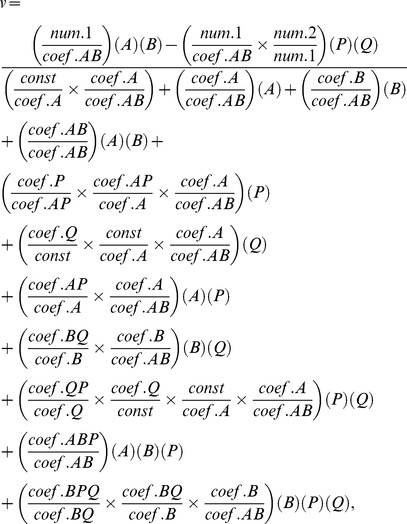
(8)where the quantities *num*, *coef*, and *constant* are parameters that govern the individual reaction steps. While complicated in detail, the format is that of the mass action law.

Outside biochemistry, mathematical modeling has been used successfully in ecology for a long time. Here, the default for describing the dynamics of two interacting species is a product of the two population sizes and a rate constant. The most prevalent systemic model of this type is the Lotka-Volterra system [33], [34], which describes the population dynamics of *n* species *i* with *P_i_* individuals, inhabiting the same space. The generic formulation of such a model is
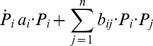
(9)It is immediately evident again that the rate of change in the prevalence of a species is a linear combination of a species-intrinsic term and of pairwise products. Specifically, the intrinsic growth or decay process of each variable *P_i_* is captured as a univariate mass action function, and the interactions between each pair of populations *P_i_* and *P_j_* follow the typical mass action law for two substrates. The structure of Lotka-Volterra models looks deceivingly simple and restrictive, but these models are capable of describing arbitrarily complex dynamics, including various types of oscillations and chaos [35]–[37].

**Figure 2:**
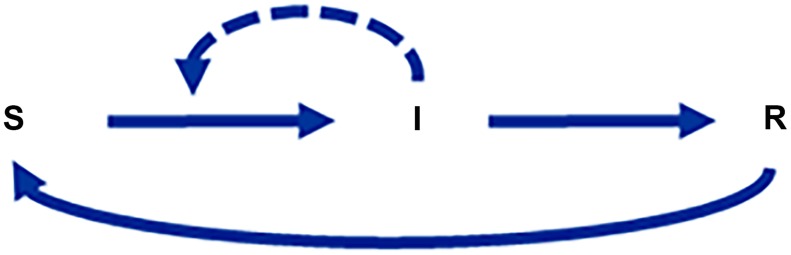
Typical prototype for a model describing the spread of an infectious disease. S, I, and R describe pools of susceptible, infected, and recovered individuals. Susceptible individuals only become sick as the result of contact with an infected person.

Mathematical epidemiologists often study the spread of an infection with some variation of the so-called SIR or SIRS model [38], which describes the dynamics of susceptible (S), infective (I), and recovered (R) individuals, as diagrammed in Fig. 2.

The corresponding equations
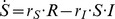


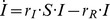
(10)

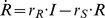
are directly in the format of the mass action law.

In the examples so far, the reaction or change rates are linear combinations of individual states (concentrations or prevalences) and their products. It is quite evident that this linearity is a stark idealization that quickly fails in realistic situations and even in simple chemical reaction systems where, for instance, diffusion limitation leads to nonlinearities. In the late 1960, Savageau initiated a long discussion about modeling biological systems in a general nonlinear, canonical manner [39]–[41]. Within the context of biochemistry, he argued that the generalized framework of Michaelis-Menten representations is impractical for modeling large biochemical systems. It also obscures insights into what is truly driving a complex system and which components are particularly sensitive or influential. Except for linear models, the only candidate for a systemic modeling structure at the time was the Lotka-Volterra formalism, which however turned out to be ill suited for representing metabolic systems (for details, see discussion in [42]).

Instead of designing patches for the existing ad hoc approaches, Savageau proposed a new framework he called Biochemical Systems Theory (BST) [31], [39]–[41] that was partially inspired by mass action functions and by Bode analysis, which is used in electrical engineering and linearizes arbitrary nonlinear functions upon logarithmic transformation in a piecewise manner [40]. Using the combination of these concepts as a starting point, Savageau proposed power-law formulations as core representations for biochemical processes. The format of this particular type of formulation is directly derived from first mathematical principles, because it is the result of a Taylor linearization, once variables and processes have been transported into a logarithmic space [40], [42]–[45]. Because of the fixed structure with which processes are represented, the steps of model design, diagnostics, and analysis follow rather strict rules, and the power-law models can therefore be considered “canonical” [44], [46].

The generic process representation in this format is
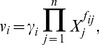
(11)where the rate constant γ*_i_* is non-negative and the kinetic orders *f_ij_* are positive for variables *X_j_* that have an augmenting or activating effect on *v_i_*, negative for variables that have an inhibiting effect, and 0 for variables that do not have a direct effect on *v_i_* at all. In other words, the kinetic-order parameters quantify the strength of the effect that a variable has on a process. For reversible processes, a linear combination of two such power expressions may be used for formation and degradation.

**Figure 3:**
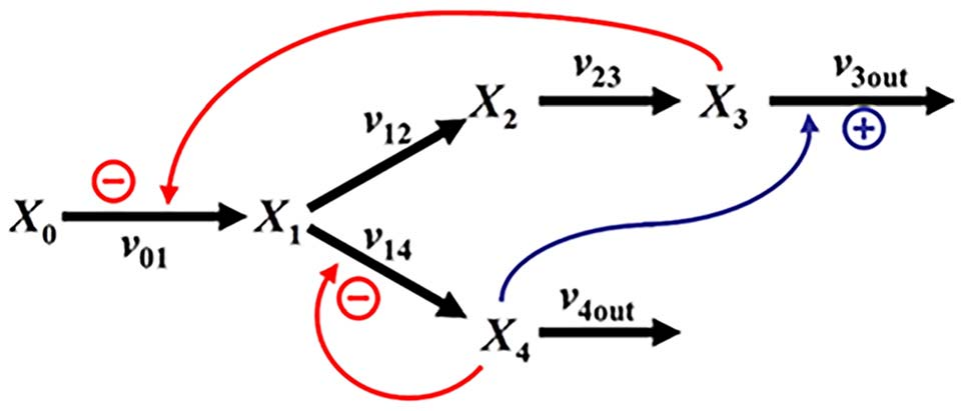
Generic pathway with one activating and two inhibitory signals. Subscripted *X*'s are metabolites, while subscripted *v*'s are processes.

A significant advantage of this power-law formulation is the fact that biochemical diagrams can immediately be converted into well-structured equations, quasi as a default representation. Specifically, each process is written as a power-law term that contains exactly those variables that directly affect this process, be it as a substrate, modulator, or regulator. As an illustration, it is easy to write down a stoichiometric model of the generic diagram in Fig. 3 where, for instance, the dynamics of *X*_1_ is directly given as
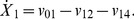
(12)However, it is not at all evident how to formulate the processes *v_ij_*. The default in biochemistry might be a Michaelis-Menten model, but it is known that its prerequisites are seldom satisfied in vivo [47]. By contrast, a power-law formulation makes no assumptions beyond positivity of its state variables and is, at the same time, mathematically guaranteed to be correct at an operating point of one's choice, and very accurate close to it, if parameter values are chosen appropriately. Thus, one can immediately formulate the dynamics of *X*_1_ as
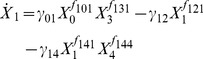
(13)and the problem of choosing a functional format is reduced to a much simpler task of estimating parameter values.

The nonlinear power-law terms in Equation 11 enter a system of differential equations in one of two ways. In the Generalized Mass Action (GMA) representation, each process is represented as one power-law term, as shown in Equations 12–13, and the result is therefore a set of differential equations whose right-hand sides each consist of a difference between the sum of power-law terms for all influxes and the sum of power-law terms for all effluxes. In the alternate S-system representation, all influxes are first collected, and their sum is represented with a single power-law function. The same is done with all effluxes, so that each S-system equation contains a single difference between two power-law terms. Thus, the second and third terms on the right-hand side of Equation 13 would be represented with a single power-law term containing *X*_1_ and *X*_4_.

The power-law formulation of process representations (Equation 11) is clearly a direct generalization of Guldberg and Waage's mass action law [3] as it is usually presented today. However, if one returns to their first article in the series [2], one finds the following, slightly different formulation of a chemical reaction: “If the amounts of the two substances affecting each other are called M and N, then the substitution force for them is α(*M^a^N^b^*). The coefficients α, *a*, and *b* are constants that, if all other conditions are kept the same, depend exclusively on the nature of the substances.” Thus, the original mass action law [2] was in power-law format with adjustable parameters. Between 1864 and 1867, the exponents *a* and *b* disappeared from the discussion, except for the case of integer stoichiometric coefficients where, for instance, two molecules of M were involved, thereby requiring *a* = 2, as we discussed before. In fact, Guldberg and Waage did not even mention the exponents anymore in the 1867 paper, stating “We have concluded from our experiments that the force [which brings forth the formation of A′ and B′ from A and B] is proportional to the product of the active masses of the bodies A and B. If we denote the active masses of A and B with *p* and *q*, and the affinity coefficient with *k*, then the force = *k*·*p*·*q*.” The authors do give somewhat of a verbal explanation: “As we have stated several times, the force *k p q* or the force between A and B is not the only force in effect during the reaction. Other forces attempt to slow down or speed up the formation of A′ and B′. Let us posit, however, that the other forces do not exist, and we see how the formulae derive in this case. We believe that the consideration of this ideal reaction…will give the reader a clear and lucent impression of our theory.” In the power-law representation (Equation 11), the real-valued kinetic order exponents account for such forces, even if they are not known to exist. These may include ill-characterized features of system components, molecular crowding, fractal kinetics, and a host of other phenomenological relationships, in a most generic and minimally biased fashion, which is guaranteed by Taylor's theorem and, at the same time, simple yet quite flexible. Linear combinations of power-law terms increase this flexibility much further.

One might note that mass action representations have been used for quite a while as the basis for developing a general theory of chemical reaction networks (CRN) [48]–[50], and it was recently demonstrated that power-law representations are beneficial extensions within this theory [51].

Intriguingly, any set of ordinary differential equations can be reformulated equivalently as a set of power-law equations, when auxiliary variables are defined [37], [52]. Thus, even complicated oscillations, chaos, and other dynamic features are within the repertoire of the power-law formalism [53], [54]. This fact is surprising and demonstrates mathematically that the power-law format is amazingly rich and that it indeed constitutes a canonical format that permits the design and analysis of models in a rigorously prescribed manner [44], [55], [56].

## Future Perspective

Guldberg and Waage posited a bold conjecture regarding the mathematical representation of chemical reactions. Although the concepts of the proposed mass action law were deduced from rather sparse data, different groups later demonstrated that the law can be derived rigorously from first principles of thermodynamics and stochastic processes for reactions that take place in ideal gases under constant temperature and pressure. Thus, Guldberg and Waage correctly solved a fundamental problem. Nonetheless, a more general question persists as a challenging task for the future, namely, how to determine appropriate representations of chemical, biochemical, and biological processes under realistic conditions, for instance, inside cells that are all but homogeneous. It is quite evident that most biological processes, although of course obeying the laws of physics, cannot realistically be formulated in terms of first physical principals, because they are driven by numerous forces, factors, and spatial aspects that are tightly intermingled, as we discussed before.

In response to this challenge, many ad hoc representations have been proposed for specific applications, but it is highly unlikely that these will be able to serve as a foundation for a general modeling framework in biology, let alone serve as a basis for theoretical analysis. For example, the widely used Michaelis-Menten models and their generalizations are not particularly well suited for theoretical investigations, because they become mathematically very cumbersome for large systems. Of special importance is the fact that they do not allow explicit steady-state computations, which renders stability and sensitivity calculations difficult [57]. The best chance for systematic theoretical and applied analyses therefore appears to rest with effective approximations, for instance, based on mass action or power-law formulations.

Nonetheless, the deeper question reemerges whether it is possible to infer truly optimal representations for complex biological processes. The search for the most appropriate representations falls into two overlapping categories. In the first case, one assumes that a valid representation structure is known and attempts to identify its optimal numerical instantiation. In other words, this first case concerns parameter optimization against experimental data. In this context, the homogenous structure of mass action and power-law representations allows interesting options. Examples are alternative regression [58] and the recently proposed method of multivariate metamodeling [59]. The former is an effective linear regression strategy for the class of S-systems. For the latter, one initially uses extensive computer simulations to establish, once and for all, a catalog of responses of individual power-law terms and their combinations. Once this catalog is available, new empirical datasets may be fitted against the “data bank” in the catalog by local interpolation. This type of meta-modeling also assists with the assessment of “neutral” or “sloppy” parameter sets, which all offer similarly good data fits [60], [61].

The second category concerns the situation where it is unknown what types of process description might be most appropriate. This category is challenging, as individual process descriptions are usually embedded in a system of differential equations, where compensation among, and even within, terms can lead to entirely wrong parameter sets, yet identify them as feasible [62]. One may anticipate that if the parameter values, and in particular the signs of the kinetic orders in a canonical power-law representation, as initially stipulated by Guldberg and Waage in 1864, can reliably be estimated from experimental data, it can help uncover the regulatory control structure of a system as a function of its position in state space [63]. Indeed, this structure would be reflected in the Jacobian architecture of the local power-law model around each experimentally investigated operating point. This knowledge of the Jacobian architecture across the state space could then serve as a tremendous guide for modeling attempts where the aim is to capture the global dynamics of a system.

## Conclusions

Over the past 150 years, the mass action law has established itself as the most used default model in mathematical modeling and systems biology, to a point where it is considered undisputed truth that needs no further explanation or justification. It was probably Guldberg, the mathematician on the team, who had the intuition to include exponents in the original 1864 rate law. His rationale is unknown, but it is clear that the exponents provided greater flexibility. The exponents were not explicitly specified, but seen as properties of the molecular species in the reaction, and were omitted later because they were apparently not needed. As an added benefit, the analysis of the simplified law of mass action was certainly easier without the exponents. Intriguingly, Guldberg's intuition reemerged rigorously almost 100 years later in the form of power-law models. These models are mathematically very flexible, as they contain additive, multiplicative, and power elements; indeed, their structure was shown to be capable of modeling any systems of differential equations [52]. The models are also very flexible from a statistical point of view, because they can represent the probabilities of events such as molecular collisions or the spread of diseases. In fact, if probability distributions are formulated as power-law models, they exhibit features of limiting distributions similar to the normal distribution [64].

In the foreseeable future, it might be fruitful to explore power-law models further as intermediates between statistical, data-driven modeling and mechanistic modeling. Statistical modeling—even if based on experimental data—views the observed system “from the outside” and lacks the ambition to describe the system mechanistically “from within” in order to specify reasons for the observed changes. By contrast, theory-driven mechanistic modeling captures why a system changes. It requires good theory or meaningful tentative ideas about the inner workings of a system and runs the risk of over-parameterization. However, it excels in combining a wide range of disjoint empirical observations, assumptions, and prior knowledge into meaningful quantitative descriptions, and can be used for assessing the validity of the prior knowledge and assumptions as well as for forecasting system responses under new circumstances. Thus, the law of mass action and its modern instantiation in power-law models is not only a useful way to think about chemical reactions but also a useful explorative data analytic tool in chemistry, biochemistry, biology, and possibly elsewhere.
